# Thioredoxin interacting protein (TXNIP) regulates tubular autophagy and mitophagy in diabetic nephropathy through the mTOR signaling pathway

**DOI:** 10.1038/srep29196

**Published:** 2016-07-06

**Authors:** Chunling Huang, Yuan Zhang, Darren J. Kelly, Christina Y. R. Tan, Anthony Gill, Delfine Cheng, Filip Braet, Jin-Sung Park, Carolyn M. Sue, Carol A. Pollock, Xin-Ming Chen

**Affiliations:** 1 Kolling Institute, Sydney Medical School Northern, University of Sydney, Royal North Shore Hospital, St Leonards, Sydney, NSW, 2065, Australia; 2 Department of Medicine, St Vincent’s Hospital, University of Melbourne, Fitzroy, VIC, 3065, Australia; 3 Department of Anatomical Pathology, Australia and Cancer Diagnosis and Pathology Group, Kolling Institute, Royal North Shore Hosptial, St Leonards, Sydney, NSW, 2065, Australia; 4 School of Medical Sciences (Discipline of Anatomy and Histology)–The Bosch Institute, University of Sydney,Sydney, NSW, 2006, Australia; 5 Australian Centre for Microscopy & Microanalysis, Madsen Building, University of Sydney, Sydney, NSW, 2006, Australia; 6 Department of Neurogenetics, Kolling Institute, Northern Sydney Local Health District, Sydney Medical School - Northern, University of Sydney, St Leonards, Sydney, NSW, 2065, Australia

## Abstract

Hyperglycemia upregulates thioredoxin interacting protein (TXNIP) expression, which in turn induces ROS production, inflammatory and fibrotic responses in the diabetic kidney. Dysregulation of autophagy contributes to the development of diabetic nephropathy. However, the interaction of TXNIP with autophagy/mitophagy in diabetic nephropathy is unknown. In this study, streptozotocin-induced diabetic rats were given TXNIP DNAzyme or scrambled DNAzyme for 12 weeks respectively. Fibrotic markers, mitochondrial function and mitochondrial reactive oxygen species (mtROS) were assessed in kidneys. Tubular autophagy and mitophagy were determined in kidneys from both human and rats with diabetic nephropathy. TXNIP and autophagic signaling molecules were examined. TXNIP DNAzyme dramatically attenuated extracellular matrix deposition in the diabetic kidneys compared to the control DNAzyme. Accumulation of autophagosomes and reduced autophagic clearance were shown in tubular cells of human diabetic compared to non-diabetic kidneys, which was reversed by TXNIP DNAzyme. High glucose induced mitochondrial dysfunction and mtROS production, and inhibited mitophagy in proximal tubular cells, which was reversed by TXNIP siRNA. TXNIP inhibition suppressed diabetes-induced BNIP3 expression and activation of the mTOR signaling pathway. Collectively, hyperglycemia-induced TXNIP contributes to the dysregulation of tubular autophagy and mitophagy in diabetic nephropathy through activation of the mTOR signaling pathway.

Hyperglycemia activates various pathways to induce oxidative stress, pro-fibrotic factors, advanced glycation end-products and activation of the renin-angiotensin-aldosterone system, leading collectively to renal injury, excessive extracellular matrix production and albuminuria^1^. The resultant alterations in energy utilization and mitochondrial function are considered to play critical roles in the initiation and development of diabetic nephropathy^2^. Thioredoxin-interacting protein (TXNIP) as a natural inhibitor of thioredoxin is an early-response gene which is markedly induced by hyperglycemia. It evokes a program of cellular defense/survival mechanisms that ultimately lead to oxidative stress, endoplasmic reticulum stress/inflammation, autophagy, and apoptosis^3^,^4^. TXNIP has been recognized as a key regulator of pancreatic β-cell biology, with increased expression of TXNIP in β-cells inducing β-cell apoptosis. Conversely, TXNIP deficiency protects against both type 1 and type 2 diabetes by promoting β-cell survival^5^. In diabetic kidneys, increased TNXIP expression is associated not only with increased oxidative stress but also with excessive matrix production that characterizes diabetic kidney disease^6^. A recent report has confirmed TXNIP deficiency protects against the development of diabetic nephropathy^7^.

Autophagy is a highly conserved intracellular degradation system by which cells degrade and recycle macromolecules and organelles. Dysregulation of autophagy is implicated in the pathogenesis of various renal diseases including diabetic nephropathy, and targeting the autophagic pathway to activate and restore autophagy may be renoprotective^8^,^9^. We have previously shown that inhibition of TXNIP using DNAzyme technology attenuated oxidative stress, inflammasome signaling, tubulo-interstitial fibrosis and collagen deposition in the tubulo-interstitium of diabetic rats^10^, and hyperglycemia led to dysfunctional autophagy in renal tubular cells with decreased autophagic clearance^11^. However, the mechanistic link between TXNIP and autophagy remains to be clarified. In addition, mitophagy, the selective degradation of mitochondria by autophagy, serves to eliminate the subset of mitochondria that over-produce reactive oxygen species, thus reducing the oxidative burden. However, the regulation of mitophagy in renal tubular cells has not been clearly understood. In this study we show that increased expression of TXNIP in the diabetic kidney and proximal tubular cells exposed to high glucose contributes to dysfunctional autophagy and mitophagy through activation of the mTOR signaling pathway, while inhibition of TXNIP functionally improves autophagy and mitophagy in diabetic nephropathy.

## Results

### Inhibition of TXNIP attenuated diabetic-induce renal interstitial collagen deposition and overexpression of type I collagen in diabetic rats

**Figure 1:**
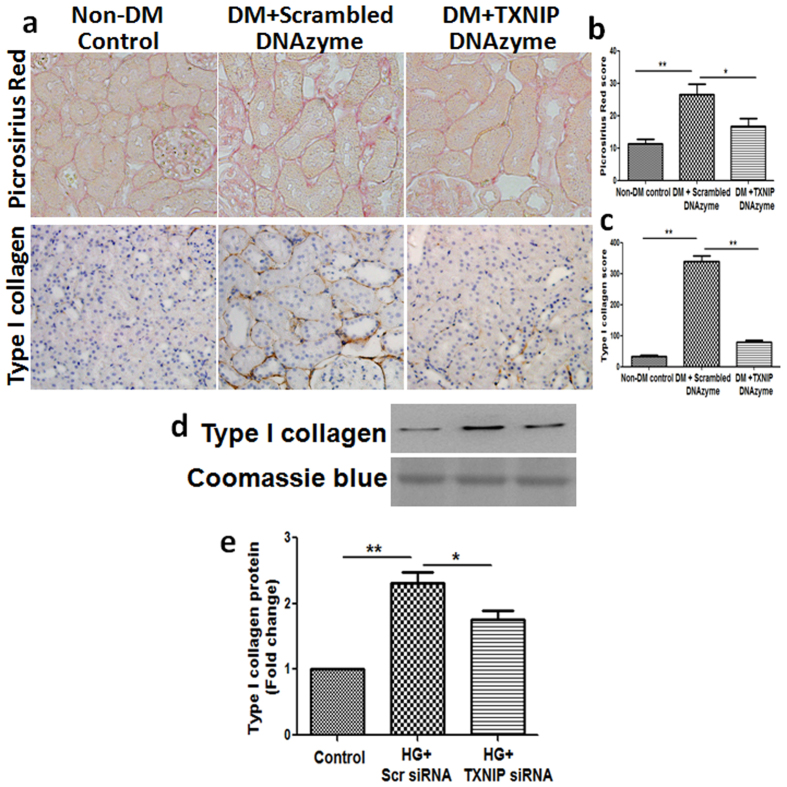
Inhibition of TXNIP attenuated diabetic-induce renal interstitial collagen deposition and overexpression of type I collagen in diabetic rats. (mRen-2) 27 rats were injected with streptozotocin (STZ) to induce diabetes or citrate buffer alone as non-diabetic control. Kidney tissues were collected for immnostaining at 12 weeks after the induction of diabetes. (**a**) Representative images show picrosirius red and immunohistochemical staining of type I collagen in the renal cortex from non-diabetic controls (non-DM control), diabetic rats treated with scrambled DNAzyme (DM + Scrambled DNAzyme) and diabetic rats treated with TXNIP DNAzyme (DM + TXNIP DNAzyme). N = 6. Quantitation of the degree of tubulointerstitial injury (**b**) and type I collagen (**c**). HK2 cells transfected with scrambled siRNA or TXNIP specific siRNA were treated with high glucose for 48 hours. (**d**) Western blot analysis showed that TXNIP siRNA inhibited high glucose-induced type I collagen expression in HK2 cells. (**e**) Quantification of type I collagen expression in high glucose-induced HK2 cells. Results are presented as mean ± SEM. *P < 0.05 and **P < 0.01. N = 4. Original magnification: ×200.

To determine whether TXNIP is involved in the pathophysiology of diabetes nephropathy, we examined the effect of modifying TXNIP expression on the development of interstitial fibrosis by measuring interstitial collagen fibril deposition using picrosirius red staining. Animals with diabetes mellitus demonstrated a marked increase in renal interstitial collagen deposition (*P* < 0.01, Fig. 1a), while inhibition of TXNIP with DNAzyme significantly reduced diabetes-induced excessive matrix deposition (*P* < 0.05, Fig. 1b).

To further determine that TXNIP is involved in diabetic renal fibrosis, we evaluated the effect of TXNIP on the expression of type I collagen, one of the predominant collagens in diabetic interstitial fibrosis^12^. Immunohistochemical staining showed that diabetes mellitus resulted in significantly increased expression of type I collagen (*P* < 0.01, Fig. 1a) localized to the interstitial areas of diabetic kidneys, which was significantly attenuated by the administration of TXNIP DNAzyme (*P* < 0.01, Fig. 1c). This result was further validated in the *in vitro* model; Human proximal tubular cells (HK2 cells) transfected with scrambled siRNA or TXNIP siRNA were concurrently exposed to high glucose. As expected, exposure of HK2 cells to high glucose resulted in significantly increased expression of type I collagen compared with the control, while concurrent exposure to TXNIP siRNA inhibited high glucose-induced increases in type I collagen (*P* < 0.05, Fig. 1d,e). These results indicate that TXNIP is involved in excessive extracellular matrix production that characterizes renal interstitial fibrosis in diabetic nephropathy.

We have previously demonstrated that TXNIP DNAzyme treatment significantly attenuated diabetes-induced increases in type IV collagen deposition in diabetic rats^10^. Collectively, these results indicate that blockade of TXNIP suppressed the production of interstitial collagens and reduced renal interstitial fibrosis in diabetic nephropathy.

### Inhibition of TXNIP attenuated diabetes-induced upregulation of LC3 and P62 expression in the renal tubule cells of diabetic rats

**Figure 2:**
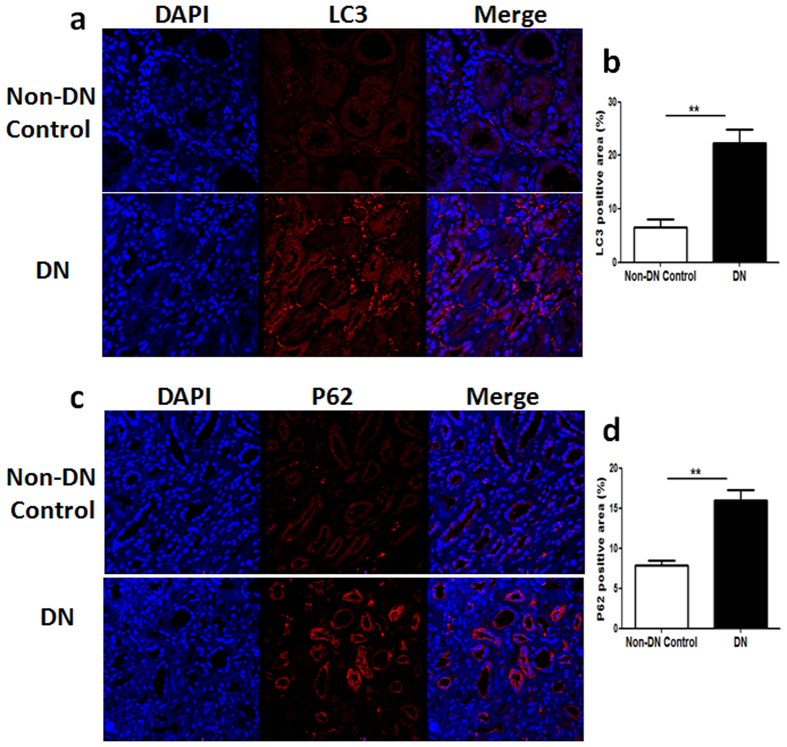
LC3 and P62 expression were increased in the renal tubular cells of the patients with diabetic nephropathy. Immunofluorescence staining showed increased expression of LC3 (**a**) and P62 (**c**) in kidney biopsies from the patients with diabetic nephropathy (DN) compared with the non-diabetic control kidneys (Non-DN control). Quantitation of LC3 (**b**) and P62 (**d**) expression in human biopsies. Results are presented as mean ± SEM. **P < 0.01. N = 6. Original magnification: ×600.

To determine whether inhibition of TXNIP attenuates diabetic renal fibrosis via regulating tubular autophagy, we first tested whether autophagy is involved in the pathogenesis of diabetic nephropathy. The autophagy marker LC3 was assessed in kidney biopsies from the patients with diabetic nephropathy and non-diabetic controls using immunofluorescence analysis. As shown in Fig. 2a, increased staining for LC3 was observed in the proximal tubular cells of diabetic kidneys, whereas only a basal level of LC3 expression occurred in the non-diabetic controls (*P* < 0.01, Fig. 2a,b). LC3 can accumulate either due to increased upstream autophagosome formation or impaired downstream autophagosome-lysosome fusion. To distinguish between these two possibilities, autophagosome clearance (also known as autophagy flux) was examined using P62, a selective substrate of autophagy. Consistent with the LC3 findings, immunofluorescence staining results showed that P62 staining was significantly increased in the tubules cells of kidney biopsies from patients with diabetic nephropathy as compared to the non-diabetic controls (*P* < 0.01, Fig. 2c,d). The increase in both LC3 and P62 expression in the renal tubule cells of kidney biopsies from patients with diabetic nephropathy demonstrated that the accumulation of autophagosomes is likely due to the suppression of autophagosome clearance, suggesting that tubular autophagy is inhibited in diabetic nephropathy.

**Figure 3:**
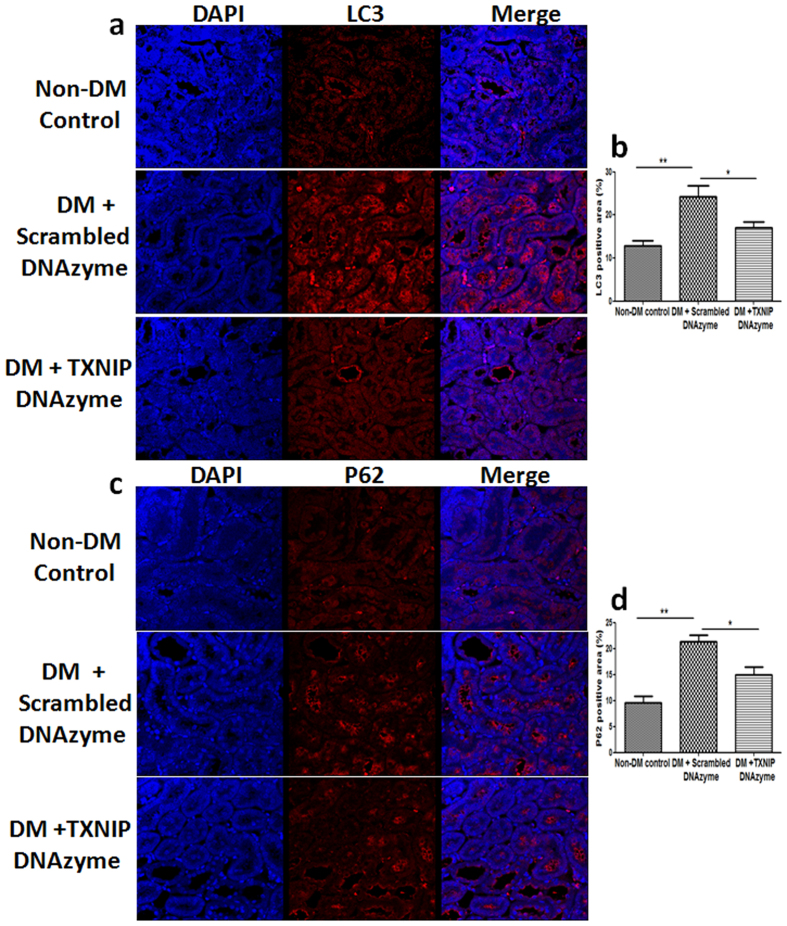
Inhibition of TXNIP attenuated diabetes-induced upregulation of LC3 and P62 expression in the renal tubule cells of diabetic rats. (mRen-2) 27 rats were injected with STZ to induce diabetes or citrate buffer alone as non-diabetic control. Kidney tissues were collected for immnostaining at 12 weeks after the induction of diabetes. Immunofluorescence staining showed that TXNIP DNAzyme treatment attenuated diabetes-induced upregulation of LC3 (**a**) and P62 (**c**) expression in the renal tubule cells of diabetic rats. Quantification of LC3 (**b**) and P62 (**d**) expression in rat kidney tissues. Results are presented as mean ± SEM. *P < 0.05 and **P < 0.01. N = 6. Original magnification: ×600.

We next determined the expression of LC3 and P62 expression in diabetic rats treated with scrambled DNAzyme or TXNIP DNAzyme. As shown in Fig. 3a, significantly increased LC3 expression was found in the renal tubule cells of diabetic rats treated with scrambled DNAzyme compared to the non-diabetic rats. Inhibition of TXNIP with DNAzyme significantly attenuated diabetes-induced upregulation of LC3 expression in the renal tubule cells of diabetic rats (*P* < 0.05, Fig. 3b). Similarly, immunofluorescence staining showed that P62 staining was significantly increased in the renal tubule cells of diabetic rat when compared to the non-diabetic controls (*P* < 0.01, Fig. 3c), which was attenuated by TXNIP DNAzyme treatment (*P* < 0.05, Fig. 3d). Collectively, these results indicate that inhibition of TXNIP attenuates diabetes-induced inhibition of tubular autophagy in diabetic rat kidneys.

### Inhibition of TXNIP reversed high glucose-induced mitochondrial dysfunction in HK2 cells

**Figure 4:**
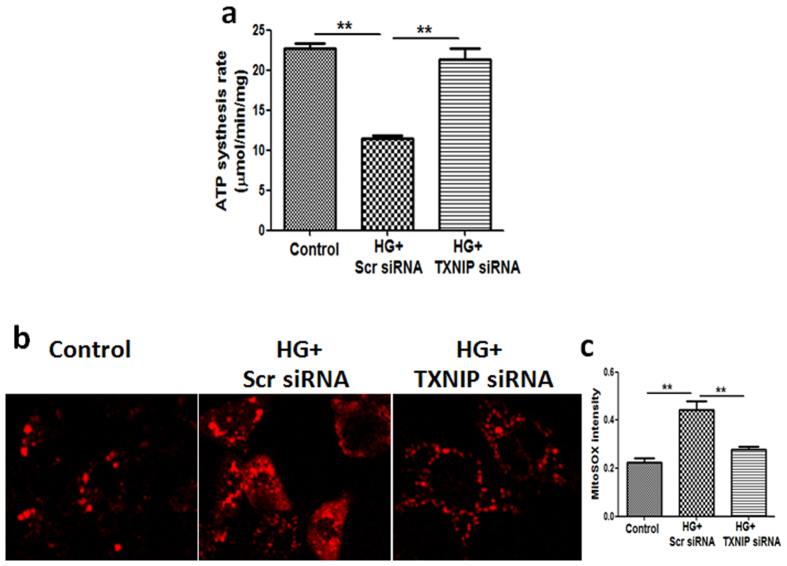
Inhibition of TXNIP reversed high glucose-induced mitochondrial dysfunction in HK2 cells. HK2 cells transfected with scrambled siRNA or TXNIP siRNA were treated with high glucose for 48 hours. (**a**) Mitochondrial function was assessed by ATP production rate. Treatment of HK2 cells with TXNIP siRNA significantly reversed high glucose-induced inhibition of mitochondrial ATP production rate. (**b**) Mitochondrial reactive oxygen species (mtROS) production was assessed by MitoSOX Red. Treatment of HK2 cells with TXNIP siRNA significantly reversed high glucose-induced mtROS production. (**c**) Quantification of MitoSOX Red fluorescence intensity in HK2 cells. Results are presented as mean ± SEM. **P < 0.01. N = 4. Original magnification: ×600.

It’s well known that oxidative stress plays an important role in the development of diabetic nephropathy^13^,^14^,^15^. Mitochondria are the major source of reactive oxygen species (ROS) and damaged/dysfunctional mitochondria are normally eliminated by autophagic degradation (i.e., mitophagy). To investigate whether TXNIP is involved in dysfunctional tubular mitophagy, mitochondrial function was firstly examined in human renal tubular cells exposed to high glucose with or without TXNIP gene silencing. Compared to the controls, mitochondrial ATP production rate was significantly lower in HK2 cells when exposed to high glucose together with scrambled siRNA (22.74 ± 0.63 for the control and 11.44 ± 0.39 for high glucose + scrambled siRNA, *P* < 0.01, Fig. 4a). Treatment of HK2 cells with TXNIP siRNA significantly reversed high glucose-induced inhibition of ATP production rate (21.35 ± 1.39, *P* < 0.01).

We then determined mitochondrial ROS (mtROS) production in HK2 cells by MitoSOX Red, a fluorogenic dye for selective detection of superoxide in the mitochondria. Application of MitoSOX Red in the untreated cells revealed a low level of fluorescence, indicating basal levels of mitochondrial ROS production (Fig. 4b). Exposure to high glucose induced a 2.45 ± 0.33 fold increase in MitoSOX Red fluorescence in HK2 cells compared to the control cells, which was inhibited by TXNIP silencing (*P* < 0.01, Fig. 4c). These data collectively demonstrate that high glucose impaired mitochondrial function through a TXNIP-related mechanism in HK2 cells and inhibition of TXNIP reversed high glucose-induced mitochondrial dysfunction.

### TXNIP gene silencing reversed high glucose-induced inhibition of mitophagy

**Figure 5:**
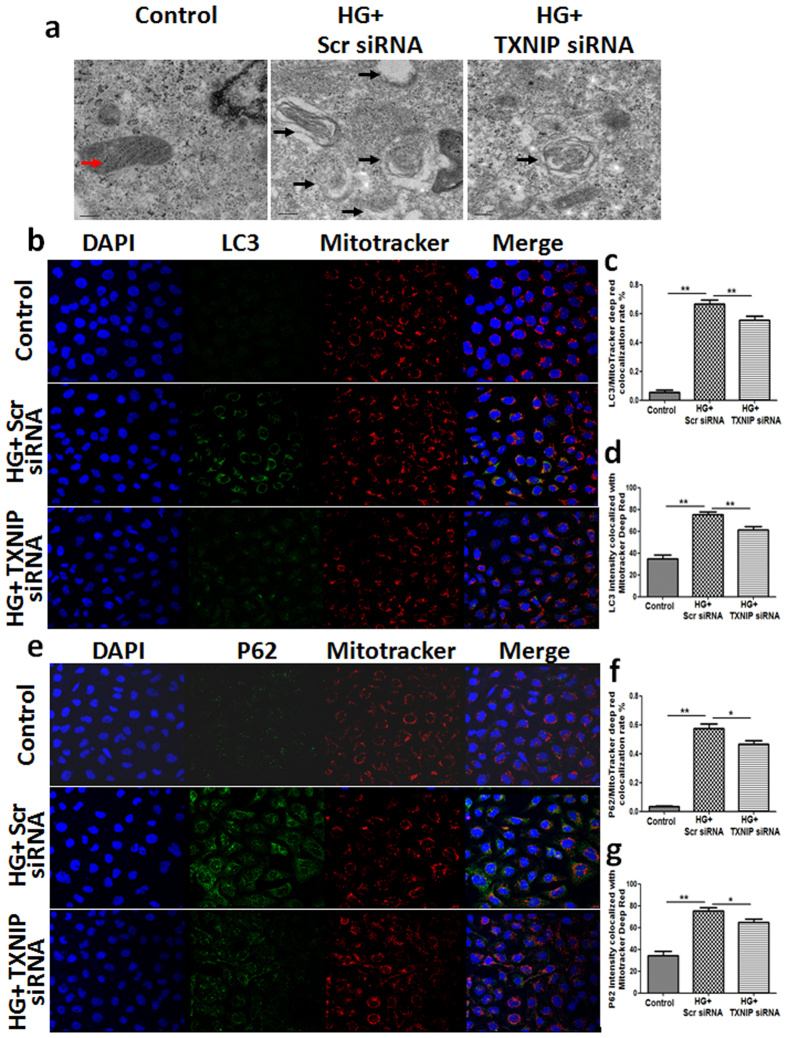
TXNIP gene silencing reversed high glucose-induced inhibition of mitophagy. HK2 cells transfected with scrambled siRNA or TXNIP siRNA were treated with high glucose for 48 hours. (**a**) Transmission electron microscopy evaluation of mitophagy in high glucose-induced HK2 cells. Representative electron micrographs show mitochondria morphology in treated HK2 cells. Red arrow indicates well developed/preserved mitochondrial structures, while black arrows show abnormalities in mitochondria morphology indicative of mitophagy with dark electron dense mitochondria lacking typical cristae or residual mitochondrial bodies present in autolysosomes. Scale bars, 0.2 μm. Confocal microscopy of MitoTracker Red-labeled mitochondria and LC3 staining (**b**) and quantification of colocalization rate (**c**) and fluorescence intensity (**d**) of LC3/MitoTracker Deep Red. Confocal microscopy of MitoTracker Red-labeled mitochondria and P62 staining (**e**) and quantification of colocalization rate (**f**) and fluorescence intensity (**g**) of P62/MitoTracker Deep Red in HK2 cells. Results are presented as mean ± SEM. *P < 0.05 and **P < 0.01. N = 4. Original magnification: ×600.

To further investigate the role of TXNIP in dysfunctional tubular mitophagy, we initially used TEM to monitor the morphology of mitochondria. Control HK2 cells showed intact mitochondria, with well-developed cristae (Fig. 5a). Exposure of HK2 cells to high glucose revealed an abundance of swollen mitochondria, with evidence of severely disrupted cristae which were attenuated by TXNIP siRNA gene silencing.

Mitochondrial autophagy was further detected by co-localization of LC3 and P62 with MitoTracker Deep Red-stained mitochondria. As shown in Fig. 5b, the intensity of LC3 that colocalized with mitochondria was significantly increased in HK2 cells exposed to high glucose when compared to the control, which was significantly attenuated by TXNIP silencing (*P* < 0.01, Fig. 5c,d). Similarly, treatment with TXNIP siRNA significantly suppressed high glucose-induced increased colocalization and staining intensity of the MitoTracker Deep Red and P62. (*P* < 0.05, Fig. 5e–g). These data indicate that inhibition of TXNIP reversed high glucose-induced inhibition of mitophagy in HK2 cells.

### Inhibition of TXNIP suppressed diabetes-induced upregulation of BNIP3 expression in HK2 cells and the renal tubule cells of diabetic rats

**Figure 6:**
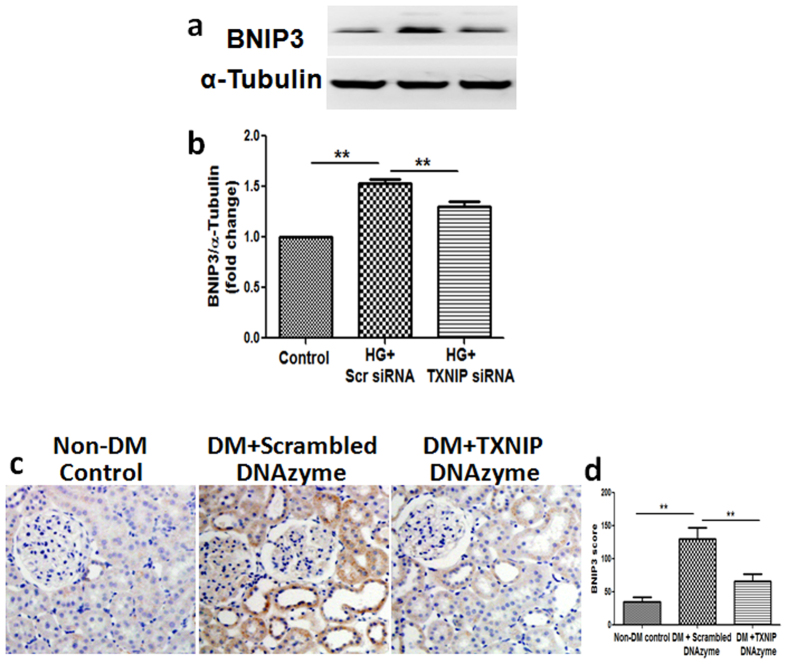
Inhibition of TXNIP suppressed diabetes-induced upregulation of BNIP3 expression in HK2 cells and the renal tubule cells of diabetic rats. HK2 cells transfected with scrambled siRNA or TXNIP specific siRNA were treated with high glucose for 48 hours. (**a**) Western blot analysis showed that TXNIP siRNA inhibited high glucose-induced BNIP3 expression in HK2 cells. (**b**) Quantification of BNIP3 in high glucose-induced HK2 cells. N = 4. (**c**) Representative images show immunohistochemical staining of BNIP3 in the renal cortex from non-DM control, diabetic rat treated with scrambled DNAzyme (DM + Scrambled DNAzyme) and diabetic rats treated with TXNIP DNAzyme (DM + TXNIP DNAzyme). N = 6. (**d**) Quantitation of BNIP3 expression in rat kidney tissues. Results are presented as mean ± SEM. **P < 0.01. Original magnification: ×200.

To further investigate the mechanism whereby TXNIP regulates mitophagy, the mitophagy regulator protein, BNIP3 was examined in HK2 cells exposed to high glucose. As shown in Fig. 6a, high glucose significantly increased the expression of BNIP3 in HK2 cells that was attenuated by TXNIP silencing (*P* < 0.01, Fig. 6b). Furthermore, we examined the expression of BNIP3 in diabetic rat kidneys. Immunohistochemical staining confirmed a marked induction of BNIP3 in the kidneys of diabetic rats treated with scrambled DNAzyme when compared to the non-diabetic controls, which was attenuated in the kidneys of diabetic rats treated with TXNIP DNAzyme (*P* < 0.01, Fig. 6c,d). Together, these results suggest that TXNIP mediates dysregulation of tubular mitophagy with associated upregulation of BNIP3.

### Inhibition of TXNIP suppressed high glucose-induced activation of the mTOR signaling pathway

**Figure 7:**
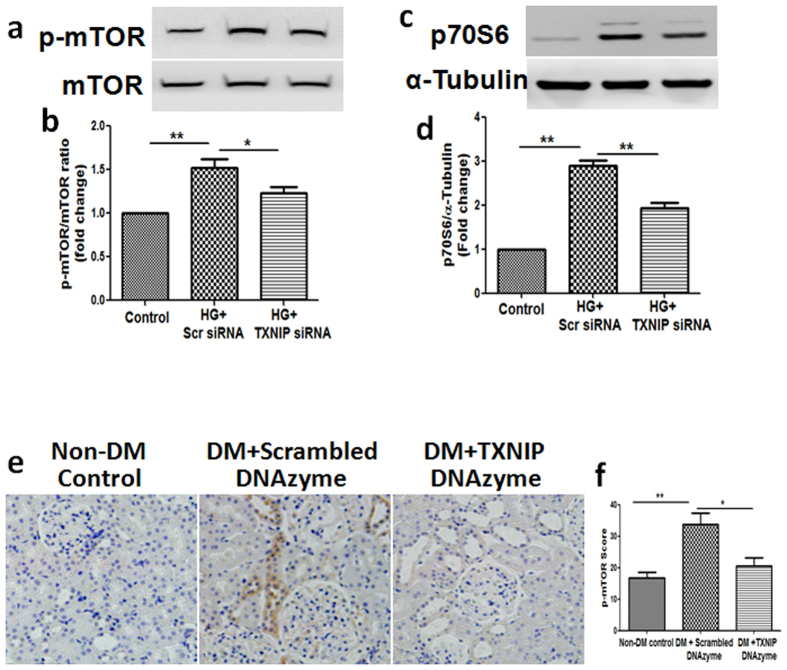
Inhibition of TXNIP suppressed high glucose-induced activation of the mTOR signaling pathway. HK2 cells transfected with scrambled siRNA or TXNIP specific siRNA were treated with high glucose for 48 hours. Western blot analysis showed that TXNIP siRNA inhibited high glucose-induced phosphorylation of mTOR (**a**) and p70S6 (**c**) in HK2 cells. Quantification of phosphorylation of mTOR (**b**) and p70S6 (**d**) in the high glucose-treated HK2 cells. N = 4. (**e**) Representative images show immunohistochemical staining of phosphorylation of mTOR in the renal cortex from non-DM control, diabetic rats treated with scrambled DNAzyme (DM + Scrambled DNAzyme) and diabetic rats treated with TXNIP DNAzyme (DM + TXNIP DNAzyme). (**f**) Quantitation of phosphorylation of mTOR expression in rat kidney tissues. N = 6. Results are presented as mean ± SEM. *P < 0.05 and **P < 0.01. Original magnification: ×200.

The mTOR signaling pathway is the major pathway regulating autophagy. To elucidate the molecular mechanisms whereby inhibition of TXNIP restores functional autophagy and mitophagy, we examined the effects of TXNIP gene silencing on the mTOR signaling pathway in HK2 cells by assessing the phosphorylation of mTOR and p70S6, the downstream target of mTOR, using western blot analysis. Exposure of HK2 cells to high glucose resulted in significantly increased phosphorylation of mTOR and p70S6 (*P* < 0.01, Fig. 7a–d) while co-incubation of HK2 cells with TXNIP siRNA suppressed high glucose-induced activation of mTOR and p70S6 (*P* < 0.05, Fig. 7a–d). Furthermore, we examined the phosphorylation of mTOR in diabetic rat kidneys. Immunohistochemical staining showed that mTOR signals were strongly activated in the kidneys of diabetic rats compared to the non-diabetic control rats (*P* < 0.01, Fig. 7e), while mTOR activation was inhibited in diabetic rats treated with TXNIP DNAzyme (*P* < 0.05, Fig. 7f).

**Figure 8:**
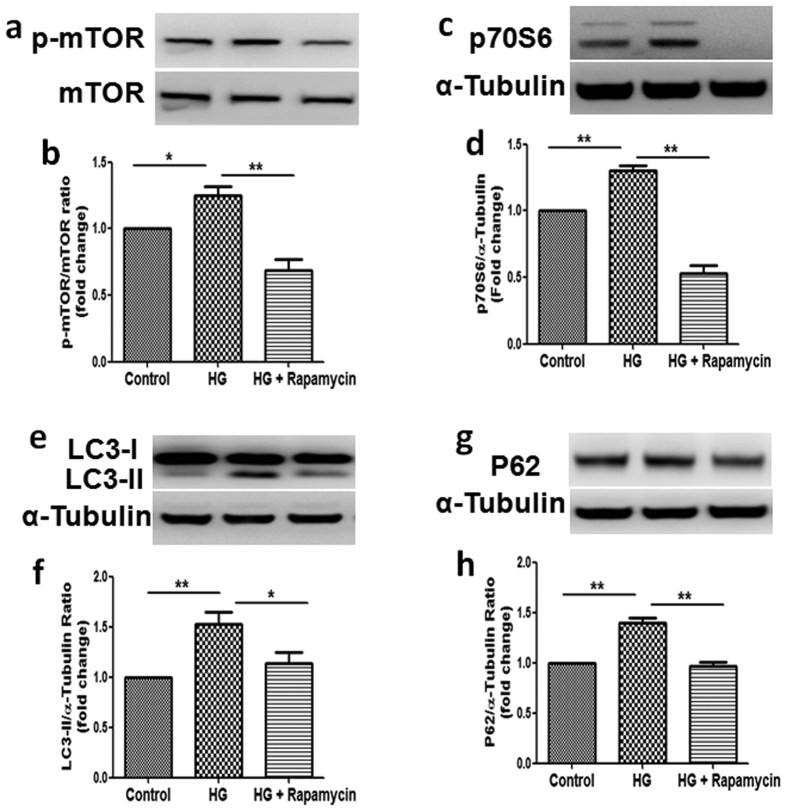
Inhibition of mTOR by Rapamycin suppressed high glucose-induced upregualtion of LC3 and P62 in HK2 cells. To evaluate the effects of mTOR inhibition, HK2 cells were exposed to high glucose (30 mM) with or without rapamycin (100 nm) for 48 hours. Western blot analysis showed that rapamycin inhibited high glucose-induced phosphorylation of mTOR (**a**) and p70S6 (**c**), expression of LC3 (**e**) and P62 (**g**) in HK2 cells. Quantification of expression of mTOR (**b**) and p70S6 (**d**), LC3 (**f**) and P62 (**h**) in HK2 cells. Results are presented as mean ± SEM. *P < 0.05 and **P < 0.01. N = 4.

To further determine the functional significance of mTOR activation on high glucose-induced inhibition of autophagy, we then tested the effect of high glucose on autophagy in the presence of rapamycin, a specific inhibitor of mTOR signaling. As shown in Fig. 8a,b, co-incubation with rapamycin significantly suppressed high glucose-induced activation of mTOR (*P* < 0.05), which was accompanied by the concomitant inhibition of p70S6 (Fig. 8c,d, *P* < 0.01). In addition, treatment with rapamycin inhibited high glucose-induced upregulation of LC3 and P62 expression (*P* < 0.05, Fig. 8e–h). These data confirm that TXNIP mediates diabetes-induced dysfunction of tubular autophagy through the mTOR signaling pathway.

## Discussion

The present study confirms that inhibition of TXNIP by delivery of specific DNAzyme reverses the excessive extracellular matrix deposition characteristic of diabetic nephropathy. The *in vivo* findings were complemented by the results from *in vitro* studies using proximal tubular cells, confirming that TXNIP siRNA mediated gene silencing normalized high glucose-induced type I collagen overexpression. We recently demonstrated that TXNIP mediates dysfunctional autophagy in renal tubular cells exposed to high glucose^11^. However, the mechanisms whereby this occurs have not been elucidated. In the current study, we have defined the role of TXNIP in regulating tubular autophagy and mitophagy in diabetic nephropathy. Our results demonstrated that tubular autophagy is dysfunctional, as indicated by increased LC3 and P62 in kidneys of both human and rats with diabetic nephropathy, and that the dysregulated LC3 and P62 expression were normalised in the kidneys of diabetic rats treated with TXNIP DNAzyme. Moreover, the *in vitro* studies have shown that high glucose induced dysregulation of tubular mitophagy in proximal tubular cells and TXNIP siRNA restored tubular mitophagy through inhibition of the mTOR signaling pathway.

It has been well documented that impaired or dysregulated mitochondrial function is associated with aging and many diseases including diabetic nephropathy^16^,^17^. When mitochondrial ATP production rate was used to examine mitochondrial function^18^ and MitoSOX Red to detect mtROS production in renal tubular cells^19^, our results showed that high glucose suppressed ATP production and induced mtROS production, which were reversed by TXNIP gene silencing (Fig. 4). Although reliable quantitative methods for specifically monitoring mitophagy in mammalian cells are not yet available, transmission electron microscopy remains one of the best approaches to provide direct evidence for mitophagy, while detection of colocalization of mitochondria and the autophagy marker LC3, has been frequently used to assess mitophagy using fluorescence microscopy^20^. Our results have shown that high glucose exposure led to abnormal morphology of mitochondria by TEM and increased accumulation of colocalized LC3 and P62 with mitochondria by immunofluorescence staining in renal tubular cells, which were reversed by TXNIP gene silencing (Fig. 5).

Autophagic removal of damaged mitochondria requires two steps: induction of general autophagy and priming of damaged mitochondria for selective autophagic recognition, mediated either by the Pink1-Parkin signaling pathway or mitophagic receptors including BNIP3^21^. BNIP3 localizes to the outer mitochondrial membrane, where it functions in mitophagy and mitochondrial dynamics, with the transmembrane domain of BNIP3 being required for mitochondrial targeting and proapoptotic functions^22^. Our data showed increased BNIP3 in renal tubular cells exposed to high glucose and in renal tubular cells of diabetic rats, which was partially attenuated by TXNIP siRNA or TXNIP DNAzyme respectively (Fig. 6).

mTOR is the master regulator of cellular metabolism, regulating cell growth and proliferation in response to a wide range of cues. Its signaling pathway is known to be dysregulated in many human diseases including diabetic nephropathy^23^,^24^. It is also well accepted that mTOR plays a crucial role in regulating autophagy^25^,^26^. In our *in vitro* studies, high glucose increased phosphorylation of mTOR in renal tubular cells, which was attenuated by TXNIP siRNA transfection or concurrent exposure to the mTOR inhibitor rapamycin. Similar beneficial effects were seen using TXNIP DNAzyme in diabetic rats (Figs 7 and 8).

We have previously reported that TXNIP was up-regulated in both *in vivo* and *in vitro* models of diabetic nephropathy and high glucose increased both TXNIP expression and its gene promoter activity independent of transforming growth factor-beta1 (TGF-β1) in renal tubular cells^27^. We have also shown significantly higher levels of TXNIP expression in the kidneys from patients and animal models with diabetic nephropathy^6^. TXNIP mediates high glucose-induced impairment of thioredoxin activity, and knockdown of TXNIP abrogated both glucose-induced collagen production and oxidative stress^6^. In addition, our studies have shown that TXNIP DNAzyme significantly attenuated the elevated renal cortical TXNIP gene and protein expression and the downstream markers of TXNIP activity such as oxidative stress, inflammasome signaling, tubulo-interstitial fibrosis and collagen deposition in the kidneys of diabetic rats compared to the diabetic control rats^10^. Recently, Shah *et al.* have reported confirmatory findings that diabetic TXNIP^−/−^ mice are resistant to diabetes nephropathy, with preserved renal function, reduced tubulointerstitial fibrosis, expression of TGF-β1, type IV collagen, oxidative stress and inflammation compared to the diabetic TXNIP^+/+^ mice^7^. Collectively these studies have demonstrated TXNIP is an important mediator of progressive tubulo-interstitial fibrosis in diabetic nephropathy.

In conclusion, our study demonstrated that upregulation of TXNIP mediates dysfunction of tubular autophagy and mitophagy through the mTOR signaling pathway and contributes to the development of diabetic nephropathy. Kidney targeted inhibition of TXNIP may be a novel therapeutic strategy for the prevention and treatment of diabetic nephropathy.

## Materials and Methods

### Materials

Anti-LC3B, P62, type I collagen and BNIP3 antibodies were purchased from Abcam (Cambridge, MA); anti-α-tubulin antibody from Sigma (St. Louis, MO), and anti-p70S6, p-mTOR and mTOR antibodies from Cell Signaling Technology (Danvers, MA). MitoSOX Red, Mitotracker Deep Red, Alexa 488 and Alexa 633-conjugated secondary antibodies were obtained from Invitrogen (Carlsbad, CA).

### Animal studies

Six-week-old female heterozygous (mRen-2) 27 rats (St. Vincent’s Hospital Animal House, VIC, Australia) were randomly assigned to receive either 55 mg/kg of streptozotocin (STZ; Sigma, MO, USA) diluted in 0.1 M citrate buffer, pH 4.5, or citrate buffer alone (non-diabetic) by tail vein injection following an overnight fast. Sequence-specific DNAzyme for rat TXNIP (accession no. U30789 at position 967-985) labeled with 6-carboxyfluorescein (6-FAM) at the 5′-end was designed as previously described^10^: TXNIP DNAzyme (5′-CATCACCA-GGCTAGCTACAACGAGATTGAGCT-3′) and scrambled DNAzyme (5′-ACACTGGA-GGCTAGCTACAACGATGACGAGTT-3′). Twenty four-hour post-STZ injection, diabetic rats were further randomized into two groups to receive treatment with either 10 μM scrambled DNAzyme (control) in saline or 10 μM TXNIP DNAzyme. Both scrambled and TXNIP DNAzymes were delivered by implanted subcutaneous osmotic minipump (Alzet model 2006; DURECT Corporation, CA, USA). Rats were weighed and blood glucose level was determined using an AMES glucometer (Accu-check Perfoma, Roche Diagnostics, Mannheim, Germany) weekly. Diabetic rats received intraperitoneal injections of insulin (2-4 units; Humulin NPH, Eli Lilly and Co., IN, USA) twice a week. All animals were killed at week 12 of diabetes. All animal experiments were approved by the Animal Research Ethics Committee of St. Vincent’s Hospital, Melbourne, Australia. The methods were performed in accordance with the approved guidelines.

### Human kidney biopsies

Human kidney biopsy specimens from patients with diabetic nephropathy were provided from the Department of Anatomical Pathology of Royal North Shore Hospital in Sydney, Australia. Kidneys removed from patients, generally due to peripheral tumor but without known kidney disease served as controls. This study was approved by the Human Research Ethics Committee of the Royal North Shore Hospital and informed consents were obtained from all participants. The methods were carried out in accordance with the approved guidelines.

### Histology and immunostaining

Rat paraffin-embedded kidney sections were used for immunohistochemical staining. Matrix deposition within the interstitium was assessed using picrosirius red stain (Polysciences, PA). For immunohistochemistry staining, after heat retrieval, endogenous peroxidase activity was blocked by incubation in 0.3% hydrogen peroxide. Following pre-incubation with 10% protein block (Dako, CA) for 10 minutes at room temperature to prevent nonspecific binding of antibodies, the tissues were incubated overnight at 4 °C with primary antibodies against type I collagen (1:500), BNIP3 (1:200) and p-mTOR (1:200). After incubation with appropriate secondary antibodies, sections were developed with 3,3-diaminobenzidine (Dako, CA) to produce a brown color and then counterstained with haematoxylin. Positive signals in the renal cortex regions were quantified using Image J software as previously described^28^.

Human biopsies and rat frozen tissues were fixed in ice-cold acetone for 10 minutes and then washed twice with ice cold PBS. Following pre-incubation with 2% BSA in PBS for 1 hour, the tissues were incubated with primary antibodies against LC3 (1:100) and P62 (1:100) for 1 hour at room temperature. After washing with PBS, cells were incubated with Alexa Fluor 633 anti-rabbit IgG (1:1000, Invitrogen) for 40 min at room temperature. Cells were then washed with PBS and mounted with 4′,6-diamidino-2 phenylindole (DAPI)-mounting medium (Invitrogen). The immunofluorescence images were collected by confocal fluorescence microscopy (Leica Microsystems, Mannheim, Germany).

### Cell culture and TXNIP gene silencing

Immortalized human proximal tubular cells (HK2 cells) were obtained from ATCC (Manassas, VA). HK2 cells were grown in keratinocyte serum-free media (Invitrogen, CA) and used for experimental purposes at passages 5–15.

HK2 cells were transfected with either TXNIP siRNA or scrambled siRNA using Lipofectamine 2000 reagent (Invitrogen, CA) according to the manufacturer’s instructions. The targeting siRNA sequence for TXNIP is 5′-CAUCCUUCGAGUUGAAUAUTT-3′ (GenePharma, Shanghai). After overnight transfection, the cells were incubated with high glucose (30 mM) for 48 hours before cell supernatants, cell lysates and total RNA were collected for further analysis.

To evaluate the effects of mTOR inhibition, HK2 cells were exposed to high glucose (30 mM) with or without rapamycin (100 nm) for 48 hours.

### Western blot

Type I collagen was measured in cell culture supernatant and cell lysates which were prepared in RIPA buffer with protease inhibitors (Roche, Germany). Equal amount of cell lysates samples were separated by SDS-PAGE, and then transferred to Hybond ECL nitrocellulose membrane (Amersham, USA). The membranes were incubated with primary antibodies against BNIP3 (1:1000), p-mTOR (1:1000), mTOR (1:1000), p70S6 (1:500), LC3 (1:1000), P62 (1:1000) and α-Tubulin (1:10,000) at 4 °C overnight followed by incubation with appropriate HRP-conjugated secondary antibodies (Amersham, USA). The chemiluminescent signals were then developed with standard ECL technique, and the bands were quantified by densitometry using LAS-4000 Imaging System (FUJIFILM, Japan).

### Assessment of mitochondrial ATP production rate

Mitochondrial ATP production rate was determined following a previously described protocol^29^. Briefly, the cells were harvested by trypsinization before determining the total protein concentration using a BCA protein assay kit (Thermo Scientific, Rockford, IL, USA) according to the manufacturer’s instruction. Cells were diluted in a cell suspension buffer (150 mM KCl, 25 mM Tris–HCl pH 7.6, 2 mM EDTA pH 7.4, 10 mM KPO4 pH 7.4, 0.1 mM MgCl_2_ and 0.1% (w/v) BSA) at 1 mg/ml total protein. ATP production was induced by incubation of 250 μL of the cell suspension with 750 μL of substrate buffer (10 mM malate, 10 mM pyruvate, 1 mM adenosine diphosphate, 40 mg/mL digitonin and 0.15 mM adenosine pentaphosphate) for 10 min at 37 °C. Following this incubation, the reaction was stopped by addition of 450 μL of boiling quenching buffer (100 mM Tris-HCl, 4 mM EDTA pH 7.75) to a 50 μL aliquot of the reaction mixture and subsequent incubation for 2 min at 100 °C. The resulting reaction mixture was further diluted 1:10 in quenching buffer, and the quantity of ATP was measured in an FB10 luminometer (Berthold Detection Systems, Germany) using the ATP bioluminescence assay kit (Roche Diagnostics, Switzerland), according to the manufacturer’s instruction.

### Mitochondrial superoxide Assay

The generation of mitochondrial superoxide anion was detected in living cells using MitoSox Red (Molecular Probes-Invitrogen) as instructed by the manufacturer. HK2 cells plated in a 6-well plate were transfected with either TXNIP or scrambled siRNA. The treated cells were then stained with 5 μM MitoSox Red for 15 min at 37 °C. After washing extraneous dyes with warm buffer, the stained cells were visualized using confocal fluorescence microscopy (Leica Microsystems, Mannheim, Germany).

### Assessment of autophagy ultrastructure using transmission electron microscopy

HK2 cells grown on glass coverslips were transfected with TXNIP or scrambled siRNA overnight and then incubated with high glucose (30 mM) for 48 hours as described above. The samples were prepared for transmission electron microscopy (TEM) as previously reported^11^. Briefly, the cells were washed with pre-warmed PBS twice, and then fixed in 2% glutaraldehyde in PBS for 60 minutes at room temperature. Then, the cells were washed with PBS 3 times and postfixed with 1% in osmium tetroxide in PBS for 1 hour. After rinsing 3 times with distilled water, the cells were further stained with 1% tannic acid for 1 hour and then, infiltrated and embedded in Epon resin. Ultrathin sections of 70 nm were generated with an ultramicrotome (Ultracut 7, Leica Microsystems) and post-stained with 2% aqueous uranyl acetate and Reynold’s lead citrate for 10 minutes each. The cells were examined with a JEOL 2100 TEM at an accelerating voltage of 200 kV.

### Indirect Immunofluorescence

To monitor mitophagy, mitochondria in HK2 cells were stained with 1 nM of MitoTracker Deep Red FM for 15 minutes at 37 °C. After fixation and blocking, the cells were incubated with primary antibodies against LC3 (1:100) and P62 (1:100) in 2% BSA in PBS for 1 h at room temperature. After washing with PBS, the cells were incubated with Alexa Fluor-488 conjugated secondary antibodies (1:1000, Invitrogen) for 40 min at room temperature and then mounted using 4′,6-diamidino-2 phenylindole (DAPI)-mounting medium (Invitrogen). The fluorescent signals were collected by confocal fluorescence microscopy (Leica Microsystems, Mannheim, Germany). The colocalization rates and intensity of LC3/MitoTracker Deep Red and P62/MitoTracker Deep Red were analyzed by Leica Image analysis software (Leica Microsystems, Mannheim, Germany).

### Statistical analysis

Data from at least four independent experiments were expressed as mean ± SEM. Statistical analysis between two groups was performed using two-tail *t*-test. Data from multiple groups was analyzed by one-way ANOVA followed by Tukey post test. Statistical significance was determined as *P* < 0.05.

## Additional Information

**How to cite this article**: Huang, C. *et al.* Thioredoxin interacting protein (TXNIP) regulates tubular autophagy and mitophagy in diabetic nephropathy through the mTOR signaling pathway. *Sci. Rep.* **6**, 29196; doi: 10.1038/srep29196 (2016).

## References

[b1] Choudhury D., Tuncel M. & Levi M. Diabetic nephropathy–a multifaceted target of new therapies. Discov. Med. 10, 406–415 (2010). 21122472

[b2] Reidy K., Kang H. M., Hostetter T. & Susztak K. Molecular mechanisms of diabetic kidney disease. J. Clin. Invest. 124, 2333–2340 (2014). 24892707 10.1172/JCI72271 PMC4089448

[b3] Devi T. S. *et al.* TXNIP links innate host defense mechanisms to oxidative stress and inflammation in retinal Muller glia under chronic hyperglycemia: implications for diabetic retinopathy. Exp Diabetes Res. 2012, 438238 (2012). 22474421 10.1155/2012/438238 PMC3313582

[b4] Mahmood D. F., Abderrazak A., El Hadri K., Simmet T. & Rouis M. The thioredoxin system as a therapeutic target in human health and disease. Antioxid Redox Signal 19, 1266–1303 (2013). 23244617 10.1089/ars.2012.4757

[b5] Chen J., Jing G., Xu G. & Shalev A. Thioredoxin-interacting protein stimulates its own expression via a positive feedback loop. Mol. Endocrinol. 28, 674–680 (2014). 24628418 10.1210/me.2014-1041 PMC4004782

[b6] Advani A. *et al.* Expression, localization, and function of the thioredoxin system in diabetic nephropathy. J. Am. Soc. Nephrol. 20, 730–741 (2009). 19211714 10.1681/ASN.2008020142 PMC2663825

[b7] Shah A. *et al.* Thioredoxin-Interacting Protein Deficiency Protects against Diabetic Nephropathy. J. Am. Soc. Nephrol. 26, 2963–2977 (2015). 25855771 10.1681/ASN.2014050528 PMC4657825

[b8] De Rechter S. *et al.* Autophagy in renal diseases. Pediatr. Nephrol. 31, 737–752 (2016). 26141928 10.1007/s00467-015-3134-2

[b9] Ding Y. & Choi M. E. Autophagy in diabetic nephropathy. J. Endocrinol. 224, R15–30 (2015). 25349246 10.1530/JOE-14-0437 PMC4238413

[b10] Tan C. Y. *et al.* Thioredoxin-interacting protein: a potential therapeutic target for treatment of progressive fibrosis in diabetic nephropathy. Nephron 129, 109–127 (2015). 25662516 10.1159/000368238

[b11] Huang C. *et al.* Thioredoxin-interacting protein mediates dysfunction of tubular autophagy in diabetic kidneys through inhibiting autophagic flux. Lab. Invest. 94, 309–320 (2014). 24492284 10.1038/labinvest.2014.2

[b12] Zeisberg M. & Neilson E. G. Mechanisms of tubulointerstitial fibrosis. J. Am. Soc. Nephrol. 21, 1819–1834 (2010). 20864689 10.1681/ASN.2010080793

[b13] Ha H., Hwang I. A., Park J. H. & Lee H. B. Role of reactive oxygen species in the pathogenesis of diabetic nephropathy. Diabetes Res. Clin. Pract. 82 Suppl 1, S42–45 (2008). 18845352 10.1016/j.diabres.2008.09.017

[b14] Ha H. & Lee H. B. Reactive oxygen species and matrix remodeling in diabetic kidney. J. Am. Soc. Nephrol. 14, S246–249 (2003). 12874440 10.1097/01.asn.0000077411.98742.54

[b15] Kashihara N., Haruna Y., Kondeti V. K. & Kanwar Y. S. Oxidative stress in diabetic nephropathy. Curr. Med. Chem. 17, 4256–4269 (2010). 20939814 10.2174/092986710793348581 PMC3708695

[b16] Quiros P. M., Langer T. & Lopez-Otin C. New roles for mitochondrial proteases in health, ageing and disease. Nat. Rev. Mol. Cell Biol. 16, 345–359 (2015). 25970558 10.1038/nrm3984

[b17] Zhan M., Brooks C., Liu F., Sun L. & Dong Z. Mitochondrial dynamics: regulatory mechanisms and emerging role in renal pathophysiology. Kidney Int. 83, 568–581 (2013). 23325082 10.1038/ki.2012.441 PMC3612360

[b18] Brand M. D. & Nicholls D. G. Assessing mitochondrial dysfunction in cells. Biochem. J. 435, 297–312 (2011). 21726199 10.1042/BJ20110162 PMC3076726

[b19] Mukhopadhyay P., Rajesh M., Yoshihiro K., Hasko G. & Pacher P. Simple quantitative detection of mitochondrial superoxide production in live cells. Biochem. Biophys. Res. Commun. 358, 203–208 (2007). 17475217 10.1016/j.bbrc.2007.04.106 PMC2228267

[b20] Ding W. X. & Yin X. M. Mitophagy: mechanisms, pathophysiological roles, and analysis. Biol. Chem. 393, 547–564 (2012). 22944659 10.1515/hsz-2012-0119 PMC3630798

[b21] Ashrafi G. & Schwarz T. L. The pathways of mitophagy for quality control and clearance of mitochondria. Cell Death Differ. 20, 31–42 (2013). 22743996 10.1038/cdd.2012.81 PMC3524633

[b22] Ray R. *et al.* BNIP3 heterodimerizes with Bcl-2/Bcl-X(L) and induces cell death independent of a Bcl-2 homology 3 (BH3) domain at both mitochondrial and nonmitochondrial sites. J. Biol. Chem. 275, 1439–1448 (2000). 10625696 10.1074/jbc.275.2.1439

[b23] Inoki K. mTOR signaling in autophagy regulation in the kidney. Semin. Nephrol. 34, 2–8 (2014). 24485024 10.1016/j.semnephrol.2013.11.002 PMC4911697

[b24] Lieberthal W. & Levine J. S. The role of the mammalian target of rapamycin (mTOR) in renal disease. J. Am. Soc. Nephrol. 20, 2493–2502 (2009). 19875810 10.1681/ASN.2008111186

[b25] Tanaka Y. *et al.* Autophagy as a therapeutic target in diabetic nephropathy. Exp Diabetes Res. 2012, 628978 (2012). 22028701 10.1155/2012/628978 PMC3199112

[b26] Kim Y. C. & Guan K. L. mTOR: a pharmacologic target for autophagy regulation. J. Clin. Invest. 125, 25–32 (2015). 25654547 10.1172/JCI73939 PMC4382265

[b27] Qi W. *et al.* High glucose-induced thioredoxin-interacting protein in renal proximal tubule cells is independent of transforming growth factor-beta1. Am. J. Pathol. 171, 744–754 (2007). 17675577 10.2353/ajpath.2007.060813 PMC1959480

[b28] Huang C. *et al.* Blockade of KCa3.1 ameliorates renal fibrosis through the TGF-beta1/Smad pathway in diabetic mice. Diabetes 62, 2923–2934 (2013). 23656889 10.2337/db13-0135 PMC3717839

[b29] Park J. S., Koentjoro B., Veivers D., Mackay-Sim A. & Sue C. M. Parkinson’s disease-associated human ATP13A2 (PARK9) deficiency causes zinc dyshomeostasis and mitochondrial dysfunction. Hum. Mol. Genet. 23, 2802–2815 (2014). 24399444 10.1093/hmg/ddt623 PMC4014187

